# Evaluation du niveau de connaissance des patients sur la gestion du traitement par les antis vitamines K dans le service de cardiologie de Ouagadougou

**DOI:** 10.11604/pamj.2014.19.286.5411

**Published:** 2014-11-15

**Authors:** André Samadoulougou, Dangwé Temoua Naibe, Germain Mandi, Relwendé Aristide Yameogo, Elisé Kabore, Georges Millogo, Nobila Valentin Yameogo, Jonas Koudougou Kologo, Anna Thiam/Tall, Boubacar Jean Yves Toguyeni, Patrice Zabsonre

**Affiliations:** 1 Service de Cardiologie du CHU Yalgado Ouedraogo, Ouagadougou, Burkina Faso; 2 Unité de Formation et de Recherche en Science de la Santé, Université de Ouagadougou, Ouagadougou, Burkina Faso

**Keywords:** AVK, niveau de connaissance, patients, CHU-YO, vitamin K antagonists, level of knowledge, patients, CHU-YO

## Abstract

**Introduction:**

Les antivitamines K (AVK), traitement anticoagulant oral le plus largement prescrit, posent un réel problème de santé publique du fait de leur risque iatrogène. L'objectif de cette étude était de préciser le niveau de connaissance des patients sur la gestion de leur traitement par les AVK.

**Méthodes:**

Il s'est agi d'une enquête transversale descriptive réalisée au CHU-Yalgado Ouédraogo, sur une période de 03 mois : du 1er mars au 31 mai 2012. Un questionnaire a été administré aux patients bénéficiant d'un traitement AVK depuis au moins un mois.

**Résultats:**

Soixante-dix patients ont été inclus dans l'étude dont 30 hommes. L'âge moyen était de 49 ans ± 16 ans. Les cardiopathies et la maladie thromboembolique veineuse justifiant l'institution du traitement AVK étaient retrouvées respectivement dans 58,6% et 41,4% des cas. Le nom de l'AVK et la raison exacte du traitement étaient connus respectivement dans 91,4% et 67,1% des cas. Plus de la moitié des patients (68,6%) savaient que les AVK rendaient le sang plus fluide. Quarante-six patients (65,7%) citaient l'INR comme examen biologique de surveillance du traitement et seulement 28 patients (40%) connaissaient les valeurs cibles. La majorité des patients ne connaissait pas les risques encourus en cas de surdosage (72,8%) et de sous-dosage (71,4%). Une automédication par anti-inflammatoire non stéroïdien était signalée par 18 patients (25,7%). Les choux (74,3%) et la laitue (62,9%), aliments à consommer avec modération, étaient les plus cités.

**Conclusion:**

Les connaissances des patients sur la gestion des AVK étaient fragmentaires et insuffisantes pour assurer la sécurité et l'efficacité du traitement. La création d'un programme d'éducation thérapeutique sur les AVK s'avère alors nécessaire.

## Introduction

Les AVK constituent le traitement anticoagulant oral le plus largement prescrit. Les indications sont en nette progression avec la croissance démographique et surtout le vieillissement de la population. Elles sont essentiellement cardiaques (prothèse valvulaire mécanique, troubles du rythme, cardiopathie ischémique) ou liées à une maladie thromboembolique veineuse (thrombose veineuse, embolie pulmonaire) [1]. Il existe un réel problème de santé publique lié à l'utilisation des AVK. Du fait de leur index thérapeutique étroit, les AVK exposent à deux risques principaux: la thrombose et l'hémorragie. Ces risques sont tels qu'ils placent les AVK au premier rang du risque iatrogène [2]. En dépit de la standardisation de la surveillance biologique par l'International Normalised Ratio (INR) et de la meilleure définition des objectifs thérapeutiques, les complications hémorragiques constituent encore la première cause d'accidents médicamenteux [3–5].

Dans ce contexte, des recommandations de pratiques cliniques ont été diffusées [6]. Elles sont orientées vers les professionnels de santé et les patients pour aider à la gestion du traitement par les AVK. Ces recommandations rappellent les règles de bon usage des AVK et indiquent en particulier que « tout patient doit être correctement et suffisamment informé et éduqué », notamment à l'aide d'un carnet d'information et de suivi mis à la disposition des soignants et des patients [7]. De plus, dans les pays développés, des programmes d'éducation thérapeutique destinés aux patients traités par AVK ont été élaborés et ont mis en évidence une corrélation positive entre les connaissances du patient acquises lors de l'éducation thérapeutique et l'obtention et le maintien de l'INR cible [8–13]. Par contre dans les pays en voie de développement, ces programmes sont quasi inexistants. Notre étude s'est proposé, de préciser le niveau de connaissance des patients sur la gestion de leur propre traitement par les AVK dans le service de cardiologie de Ouagadougou.

## Méthodes

Il s'est agi d'une enquête transversale descriptive réalisée au Centre Hospitalier Universitaire Yalgado Ouédraogo, sur une période de 03 mois: du 1^er^ mars au 31 mai 2012. Un questionnaire a été administré au cours d'un entretien semi-dirigé par les investigateurs (médecins en spécialisation de cardiologie) et après obtention du consentement, aux patients sous traitement AVK depuis au moins un mois, ou à son accompagnant en cas de troubles de la compréhension et/ou de troubles cognitifs. Les items (n = 18) du questionnaire s'inspiraient du carnet de suivi AVK élaboré par la société française de cardiologie adaptés à notre contexte[7, 14]. Etaient également renseignés: les caractéristiques socio-démographiques des patients et leur satisfaction de l'information délivrée. En cas de connaissance insuffisante des patients sur le traitement AVK, une information complémentaire était alors donnée par les investigateurs. Les données ont été analysées avec le logiciel EPI INFO version 7. Le taux de réponse conforme pour chaque item du questionnaire (rapport du nombre de réponses conformes par rapport au nombre de réponses total) a été calculé. Le test exact de Fischer et le test de Khi^2^ ont été utilisés comme test statistique. Le seuil de signification retenu était p < 0,05

## Résultats

### Caractéristiques sociodémographiques et médicales des patients

Soixante-dix patients ont été inclus dans l'étude dont 30 hommes (43%). Dans 15 cas (21%) nous avons eu recours à l'accompagnant. L'âge moyen était de 49 ans ± 16 ans (extrêmes 19 ans et 86 ans). Cinquante-sept patients (81,4%) provenaient d'une zone urbaine et 44 patients (63%) avaient un niveau de scolarisation supérieur ou égal au secondaire. La moitié des patients (51%) avaient une durée du traitement d'au moins six mois. Cinquante-sept patients (81,4%) déclaraient avoir reçu des explications en rapport avec leur maladie. Les indications du traitement anticoagulant étaient dans 29 cas (41,4%) en rapport avec une maladie thromboembolique veineuse et dans 41 cas (58,6%) en rapport avec une cardiopathie emboligène. Les Tableau 1 et Tableau 2 nous donnent respectivement la description des caractéristiques socio-démographiques et médicales des 70 patients.

**Tableau 1:** Caractéristiques socio-démographiques des 70 patients sous AVK

	Effectifs	%
**Sexe**		
Homme	30	43
Femme	40	57
**Niveau d'instruction**		
Non scolarisé	9	12,8
Primaire	17	24,3
Secondaire	31	44,3
Supérieur	13	18,6
**Niveau socio-économique**		
Faible	20	28,6
Moyen	45	64,3
Elevé	5	7,1

**Tableau 2:** Caractéristiques médicales des 70 patients sous AVK

	Effectifs	%
**Informations reçues sur la maladie**	57	81,4
**Indications du traitement AVK**		
Cardiopathies ischémiques	12	17,1
Fibrillation atriale	5	7,4
Prothèse valvulaire	7	10
Thrombus	17	24,3
Embolie pulmonaire	13	18,3
TVP*	16	22,9
**Durée du traitement**		
[1 mois – 3 mois]	21	30
[3 mois – 6 mois]	13	18,6
[6 mois – 12 mois]	24	34,3
≥ 12 mois	12	17,1

TVP: Thrombose veineuse profonde

### Evaluation des connaissances des patients sur le traitement antivitamine K

Soixante-trois patients (90%) déclaraient avoir reçu de leur médecin traitant, des informations concernant leur traitement AVK. Le nom de l'AVK, la raison exacte et le rôle du traitement AVK étaient connus respectivement des patients dans 64 cas (91,4%), 47 cas (67,1%) et 48 cas (68,6%). L'INR a été cité comme examen biologique pour la surveillance du traitement dans 46 cas (65,7%) et dans 28 cas (40%) la valeur cible était correctement précisée. Le risque en cas de surdosage était connu par 19 patients (27,1%). Vingt-deux patients (31,4%) déclaraient avoir présenté un saignement et l'attitude correcte à adopter en cas de saignement était connue par 43 patients (61,4%). L'utilisation concomitante proscrite d'anti-inflammatoires en cas de douleur était connue par 18 patients et 54 patients signalaient aux professionnels de santé la prise d'AVK en cas de prescription d'un autre traitement. Soixante patients (85,7%) signalaient avoir modifié leur alimentation depuis l'initiation du traitement AVK et 29 patients (41,4%) étaient capables de citer au moins cinq aliments riches en vitamine K à éviter. Il s'agissait de choux dans 52 cas (74,3%), de laitue dans 44 cas (62,9%), d'épinard dans 42 cas (60%), tomate dans 41 cas (58,6%), haricot vert dans 33 cas (47,1%) et des abats dans 27 cas (36,6%). Tous les patients faisaient recours à leur médecin traitant pour l'ajustement des doses AVK dès l'obtention des résultats de l'INR et le canal téléphonique était utilisé dans 14,3% cas. Dix-huit patients (25,7%) ont déclaré un oubli de prise de leur médicament et 12 de ces patients (66,7%) réagissaient convenablement en cas d'oubli. Tous les patients disposaient d'un carnet de suivi de leur traitement AVK mais les informations présentées n'étaient pas spécifiques. Les résultats de l'évaluation des connaissances des patients sont rassemblés dans le Tableau 3. L'INR était stable dans 33 cas (N = 49) et instable dans trois cas (N = 21) lorsque la durée du traitement AVK était supérieur à six mois (p < 0,000 ; RR = 1,9 ; IC à 0,95= (1,3-2,8). Le Tableau 4 présente les facteurs associés à la connaissance du traitement AVK.

**Tableau 3:** Connaissances sur le traitement AVK par nos 70 patients

Paramètres	Effectifs	%
Informations reçues de l’équipe de soins sur le traitement AVK	63	90
Nom de l'AVK	64	91,4
Raison du traitement	47	67,1
Rôle de l'AVK	48	68,6
Examen biologique de suivi	46	65,7
Valeur cible INR	28	40
Risques en cas de surdosage	19	27,1
Risques en cas de sous dosage	20	28,6
Automédication avec AINS proscrite	18	25,7
Saignement sous AVK	22	31,4
Citer au moins 5 aliments à éviter	29	41,1
Prise du traitement à heure régulière	69	98,6
Comportement à adopter en cas d'oubli	12(18)	66,7
Disponibilité d'un carnet de suivi	70	100

**Tableau 4:** Facteurs socio-démographiques associés à la connaissance du traitement AVK

	Niveau secondaire ou plus (n = 44)	Risque relatif (intervalle de confiance à 0,95)	*P*
Raison exacte traitement (n = 47)	36	1,9 (1,2-3,1)	0,0005
Rôle exacte de l'AVK (n = 48)	35	1,6 (1,05-2,4)	0,007
Valeur cible exacte INR (n = 28)	22	2,2 (1,01-4,6)	0,01

### Evaluation de la satisfaction des patients

L'information sur le traitement AVK était jugée satisfaisante et insuffisante par les patients dans respectivement 34 cas (48,6%) et 29 cas (41,4%). Par contre, 47 patients (67,1%) étaient satisfaits de leur prise en charge de façon globale.

## Discussion

Au cours de leur hospitalisation, les patients au contact des professionnels de santé acquièrent un certain niveau de connaissance sur la gestion de leur traitement AVK. Cet apprentissage résulte, en partie des informations délivrées par les médecins et infirmiers assurant le suivi, mais aussi d'autres sources très variées (entourage, médias, lectures...). Cependant, cette éducation « informelle » s'avère insuffisante et parcellaire pour garantir une bonne sécurité pour l'usage des AVK. Notre évaluation confirme les résultats d'autres études de plus grande ampleur [10, 11, 14–19]. En effet, pour la dimension cognitive, les questions portant sur la mémorisation des informations sur le traitement AVK et sur la capacité à interpréter un résultat biologique, notamment : le nom de l'AVK (91,4%), la raison du traitement (67,1%), le rôle de l'AVK (68,6%), l'examen biologique pour la surveillance (65,7%) sont bien connus par les patients. Par contre, pour la dimension comportementale, les questions portant sur la capacité d'anticipation et de prise de décision dans les situations dites « à risques », à savoir: la valeur cible de l'INR (40%), le risque en cas de surdosage (27,1%), le risque en cas de sousdosage (28,6%) sont insuffisamment connus. Janoly-Duménil et al. [14] ont montré que 70 à 80% des patients connaissaient le nom et l'indication de leur traitement AVK, mais que moins de 25% connaissaient les risques d'un surdosage ou d'un sous dosage. De même Labrosse et al. [15] soulignent que seulement 19% des patients traités par AVK connaissaient le risque d'un surdosage alors que 61% des patients connaissaient le rôle de l'AVK.

Ces constats sont particulièrement alarmants quand on sait que la qualité et l'adaptation de l'information médicale ont une influence majeure sur l'observance thérapeutique et partant sur l'efficacité et la sécurité des soins. Ces résultats suggèrent la nécessité d'améliorer l'information donnée aux patients sous AVK. Ceci pourrait passer par la création dans notre contexte d'un programme d'éducation thérapeutique adapté aux besoins de nos patients. Léger et al [9] et Satger et al [10] en évaluant des programmes d'éducation thérapeutique des patients sous AVK, ont montré que le niveau de connaissance des patients était globalement supérieur après intervention, aussi bien sur le plan cognitif que comportemental. Une association statistiquement significative a été retrouvée entre la durée du traitement AVK (supérieure à six mois) et l'équilibre de l'INR (p < 0,0001). Ceci pourrait conforter notre hypothèse de l'amélioration du niveau de connaissances des patients au contact des professionnels de santé et partant un meilleur équilibre de l'INR. Dans notre étude, 31,4% des patients ont présenté une complication hémorragique au cours de leur traitement par AVK. L'impact de l'éducation thérapeutique menée a pu être évalué par la réduction du risque iatrogène. En effet, les patients ayant bénéficié du processus éducatif ont eu un risque quatre fois plus faible de présenter un accident hémorragique et/ou une récidive thrombotique clinique que les patients du groupe témoin [10, 20, 21]. Une autre particularité de notre étude était que tous nos patients faisaient appel à leur médecin traitant pour la modification de la posologie de leur traitement anticoagulant dès l'obtention du résultat de contrôle de leur INR. Certes les patients accordent une grande confiance à l'équipe de soins; mais, ce fait témoigne des efforts à fournir pour l'atteinte d'une autonomie de gestion des AVK par nos patients. Ceci est aussi un des objectifs visé par l'éducation thérapeutique.

## Conclusion

Les connaissances des patients sur la gestion des AVK sont parcellaires et insuffisantes. Ces lacunes peuvent constituer une limite à l'observance et à une implication optimale des patients vis-à-vis de leur traitement. L'éducation thérapeutique s'impose donc comme une nécessité tout au long du traitement, condition *sine qua non* pour assurer l'efficacité et la sécurité du traitement. Cette démarche nécessite une motivation du patient, mais aussi un investissement de temps parfois difficile à consentir par les praticiens. La création d'un programme d'éducation thérapeutique impliquant tous les intervenants du réseau de soins (cardiologues, médecins généralistes, biologistes, pharmaciens, paramédicaux, patients) et adapté à notre contexte s'avère alors nécessaire.
